# Robotic-assisted laparoscopic management of mesenteric cysts in children

**DOI:** 10.3389/fped.2022.1089168

**Published:** 2023-01-10

**Authors:** Qingjiang Chen, Shuhao Zhang, Wenjuan Luo, Duote Cai, Yuebin Zhang, Zongwei Huang, Xiaoxiao Xuan, Qixing Xiong, Zhigang Gao

**Affiliations:** Department of Pediatric General Surgery, The Children’s Hospital, Zhejiang University School of Medicine, National Center for Clinical Medicine of Children’s Health and Disease, National Children’s Regional Medical Center, Hangzhou, China

**Keywords:** mesenteric cysts, laparoscopy, robotic surgery, children, prognosis

## Abstract

**Background:**

Mesenteric cysts (MCs) are rare intra-abdominal masses in children, and laparoscopic complete cyst resection is still difficult. This study reviewed our experience in diagnosing and managing MCs at our center, focusing on the clinical characteristics of MCs and the effectiveness of robotic-assisted laparoscopic surgery.

**Methods:**

We conducted a retrospective analysis of the records of all patients diagnosed with MCs and managed with robotic-assisted laparoscopic surgery at our center between February 2021 and August 2022. We analyzed demographic characteristics, clinical manifestations, preoperative imaging data, surgical methods, postoperative complications, and final outcomes.

**Results:**

Totally, 12 consecutive patients with a mean age of 5.81 ± 3.02 years were admitted. The most common symptom was abdominal pain (58.33%). Eight patients were associated with cyst complications, including five cases of infection, two cases of volvulus, and one case of hemorrhage. The mean size of cysts was 8.39 ± 5.91 cm. The cysts were located in ileal mesentery in eight cases, lesser curvature of the stomach in two cases, and colon mesentery in two cases. Solely cyst excision was performed in eight cases, and bowel *en bloc* resection of the cyst in four cases. Robotic-assisted laparoscopic surgery was performed successfully in all patients, without conversion. The mean operation time was 106.17 ± 33.74 min. Pathological results reported lymphangioma or lymphatic malformation in all patients. Two cases of chylous leakage were treated conservatively, and no complications of peritoneal infection, anastomotic leakage, and recurrence were observed.

**Conclusions:**

Mesenteric cysts should be removed promptly once the diagnosis is confirmed to avoid cyst complications. For uncomplicated mesenteric cysts, laparoscopic cyst excision, or cyst excision with bowel resection can be effectively performed in children, especially under the robot system.

## Introduction

Mesenteric cysts (MCs) are relatively rare intra-abdominal anomalies, present in 1 per 20,000 admissions in the pediatric age group (1). The etiologies are still uncertain; developmental anomalies of the mesenteric lymphatics that fail to communicate with the remainder of the lymphatic system and the venous system make the most accepted theory (2). They can occur anywhere in the gastrointestinal tract and mesentery and may extend to the retroperitoneum. Small-bowel mesentery, especially ileal mesentery, is the most common site of MCs (1, 3). The spectrum of presentation varies widely from asymptomatic abdominal mass and nonspecific abdominal symptoms such as pain and distension to the life-threatening acute abdomen (4). Some patients may be incidentally diagnosed during routine physical or imaging examinations, and some patients are even diagnosed prenatally (5).

Surgical excision is usually recommended once the diagnosis is made (3, 5, 6). Asymptomatic cysts smaller than 5 cm can be observed closely. Once the mass enlarged during observation, became symptomatic, or associated with complications, such as infection, hemorrhage, or volvulus, complete excision of the cyst with or without bowel resection is mandated (7). Open surgery is still the most commonly performed procedure, although the laparoscopic method is becoming increasingly popular. However, most MCs are multilocular and usually have an intimate relationship with major blood vessels and visceral organs. Laparoscopic MC resection is still considered to be a technique demanding operation and is associated with a relatively high recurrence rate and conversion rate (8). There are limited reports on laparoscopic management of MCs in children in the literature, and the safety and effectiveness of laparoscopic procedure have not been well defined.

This article summarized our experience in robotic-assisted laparoscopic management of 12 patients with MCs at our center in recent years, focusing on characterizing the clinical findings of the disease and the effectiveness of robotic-assisted laparoscopic treatment in children.

## Materials and methods

### Study design and patients

A retrospective study was conducted on pediatric patients diagnosed with MCs admitted to the Children’s Hospital Zhejiang University School of Medicine between February 2021 and August 2022. Patients with MCs and treated by robotic-assisted laparoscopy were included in this study. On the contrary, patients operated with the open approach, laparoscopic method, or conservative treatment were excluded from this study. All operations were performed by two skilled surgeons qualified for robotic surgery.

Clinical data were collected for demographic characteristics, clinical manifestations, details of preoperative examination, the surgical procedure performed, intraoperative findings, histological results, short- and mid-term complications, and outcomes. The follow-up period ranged from 1 to 18 months. All data were processed using descriptive statistical methods for calculating means, standard deviations, frequencies, and percentages.

### Operation technique

The patient was placed in a supine position, and according to the location of the cysts, the operation bed was in a slight Trendelenburg position or head-up tilt position. The cystic lesion was considered the “target” organ for the robotic procedure. The camera port was placed in the middle of the umbilicus. Two operative cannulas for the robotic monopolar hook or harmonic scalpel and bipolar grasper were arranged on the vertical line between the lesion and the umbilicus, with a distance of 5–8 cm from the camera port. An assistant port was made when necessary.

After ports were placed and docking was finished, gross exploration was performed to evaluate the position and extent of the cyst and its relationship with the intestine and mesenteric vessels. Cystic fluid was aspirated before removal when the cyst was too large to interfere with surgical manipulation. A variety of methods were adopted for the complete excision of the cyst with or without bowel resection. Simple cystectomy was performed for a well-defined cyst; a segmental bowel resection was necessary for complex MCs with an intimate relationship to the neighboring bowel; skeletonization of the mesenteric vessels by stripping off the cystic wall was indicated for MCs located at the root of the mesentery and with involvement of major blood vessels. Intestinal resection and suture were completed intra-abdominally using a 4-0 absorbable knotless tissue control device, and the mesenteric defect was sutured. The resected cyst/the attached intestine was placed in a sturdy endosurgical bag and brought out through the slightly enlarged umbilical incision. Abdominal drainage after excision of the cyst was placed routinely.

## Results

Between February 2021 and August 2022, 12 cases (8 boys and 4 girls) of MCs were operated on at our center under a robotic system without a laparoscopic procedure or laparotomy. This study excluded one case of MCs complicated with infection and treated with antibiotics. The patients’ mean age was 5.81 ± 3.02 years (range, 1.52–12.92 years). Intraoperatively, cysts located in ileal mesentery were observed in eight cases, in the lesser curvature of the stomach in two cases, and in the descending colon or sigmoid colon mesentery in one case. A single cyst was found in 2 patients, while the rest 10 cases were multilocular with vesicles of various sizes. The mean diameter of cysts was 8.39 ± 5.91 cm (range, 3.5–25 cm). The cystic fluid was chylous in three patients, serous in eight patients, and hemorrhagic in one patient.

Abdominal pain was the most common presenting feature (seven cases). Two patients presented with the symptom of vomiting, one patient with abdominal distension, and two cases were found accidentally by ultrasonography. Eight cases were associated with cyst complications, including five cases of infection, two cases of volvulus, and one case of hemorrhage (Table 1). Two cases of infected cysts had antibiotic therapy initially and delayed cyst resection 1–3 months later.

**Table 1:** Detail clinical data of mesenteric cysts in children.

Age (years)	Gender	Presentations	Complications	Location	Surgical procedure	Histopathology
2.67	Male	Abdominal pain, fever	Hemorrhage	Descending colon mesentery	Cyst resection	Lymphatic malformation
6.83	Female	Vomiting	Volvulus	Ileum mesentery	Cyst excision + bowl resection	Cystic lymphangioma
1.52	Male	—		Ileum mesentery	Cyst resection	Mixed lymphangioma
5.25	Male	Abdominal pain, fever	Infection	Ileum mesentery	Cyst resection	Lymphatic malformation
7.00	Female	Abdominal pain, fever	Infection	Ileum mesentery	Cyst excision + bowl resection	Cystic lymphangioma
4.25	Male	Abdominal pain	Infection	Sigmoid colon mesentery	Cyst resection	Cystic lymphangioma
3.83	Female	Abdominal pain, fever	Infection	Ileum mesentery	Cyst excision + bowl resection	Mixed lymphangioma
4.00	Male	Abdominal distension		Lesser curvature of the stomach	Cyst resection	Cystic lymphangioma
7.17	Female	Vomiting	Volvulus	Ileum mesentery	Cyst excision + bowl resection	Lymphatic malformation
5.75	Male	—		Root of the ileum mesentery	Cyst resection	Cavernous lymphangioma
12.92	Male	Abdominal pain		Lesser curvature of the stomach	Cyst resection	Cystic lymphangioma
8.50	Female	Abdominal pain, fever	Infection	Ileum mesentery	Cyst resection	Cystic lymphangioma

**Figure 1:**
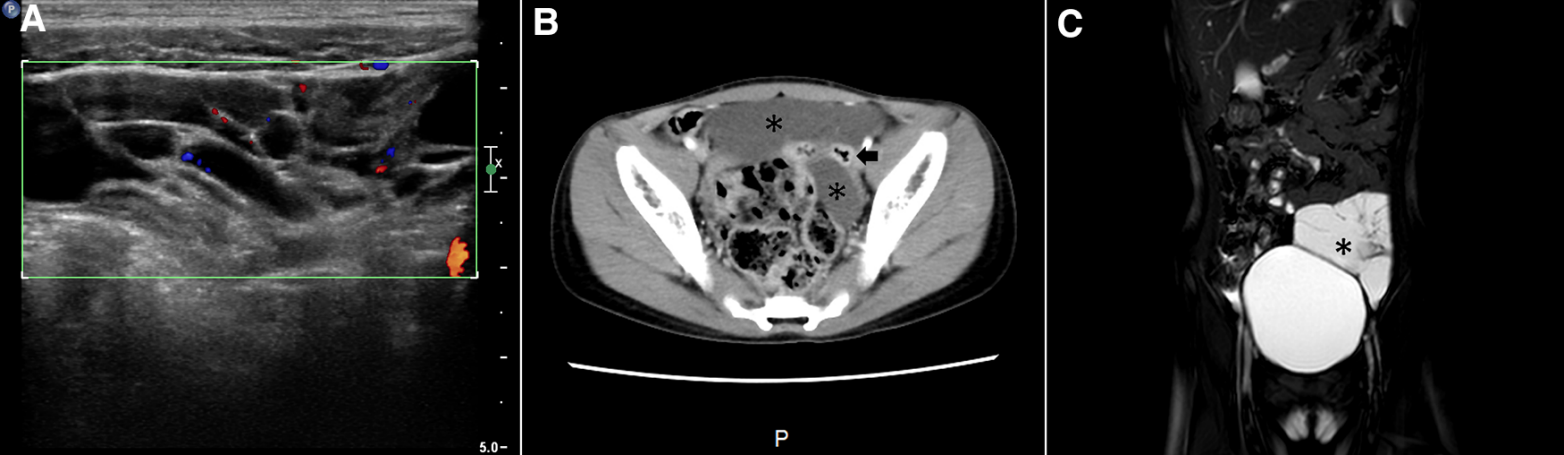
Imaging examination of a patient with a dumbbell-type ileal mesenteric cyst. (**A**) Sonography demonstrated a hypoechoic cyst with multiple internal septations; (**B**) enhanced CT scan showing a cyst (asterisk) located on both sides of the intestine (arrow), without contrast enhancement; (**C**) MRI showing an irregular cyst (asterisk) with a sharp margin and septation on a T2-weighted image.

**Figure 2:**
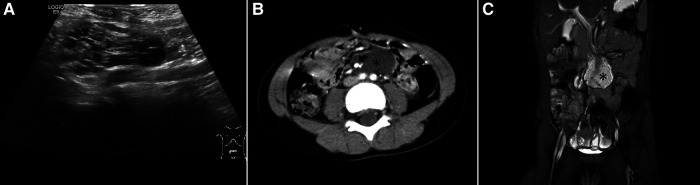
Imaging of a patient with a complicated cyst located at the root of the intestinal mesentery. (**A**) Sonograph demonstrating a hypoechoic cyst with multiple vehicles and internal septations; (**B**) enhanced CT scan showing a low-density cyst (asterisk) located at the root of the intestinal mesentery and surrounded mesenteric vessels (arrow); (**C**) MRI showing a cyst with significant signal intensities on T2-weighted imaging.

**Figure 3:**
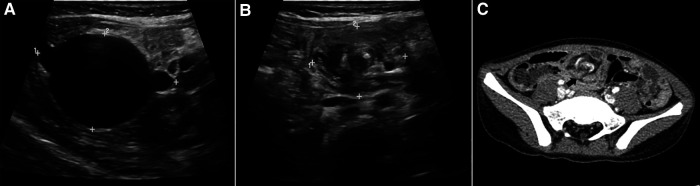
Imaging of MCs complicated with volvulus. (**A**) Sonographic imaging demonstrating a well-marginated cyst; (**B**) proximal mesentery with the alternating rings (asterisk) of low and high echogenicity due to volvulus (concentric circle sign); (**C**) enhanced CT scan revealing the whirlpool sign (asterisk) of the mesenteric artery and vein (arrow).

A variety of imaging modalities were used to confirm the diagnosis. All patients underwent ultrasonography; cysts with intra- or intercystic echogenic septa were demonstrated in 10 patients (Figures 1A, 2A), and abdominal cyst and massive ascites respectively were reported in the other two patients. Eight patients were examined by computed tomography (CT), which reported low-density cysts with no obvious enhancement and CT value ranging from 7 to 27 HU (Figures 1B, 2B). Whirlpool sign was observed in two cases and was suspected with volvulus under ultrasonic examination and CT scanning (Figures 3A–C). Nine cases were examined by magnetic resonance imaging (MRI), which demonstrated significant signal intensities on T2-weighted MRI and intracystic septations in six cases (Figures 1C, 2C). In total, 10 cases of MCs, 1 case of an omental cyst, and 1 case of an abdominal cyst were diagnosed preoperatively.

**Figure 4:**
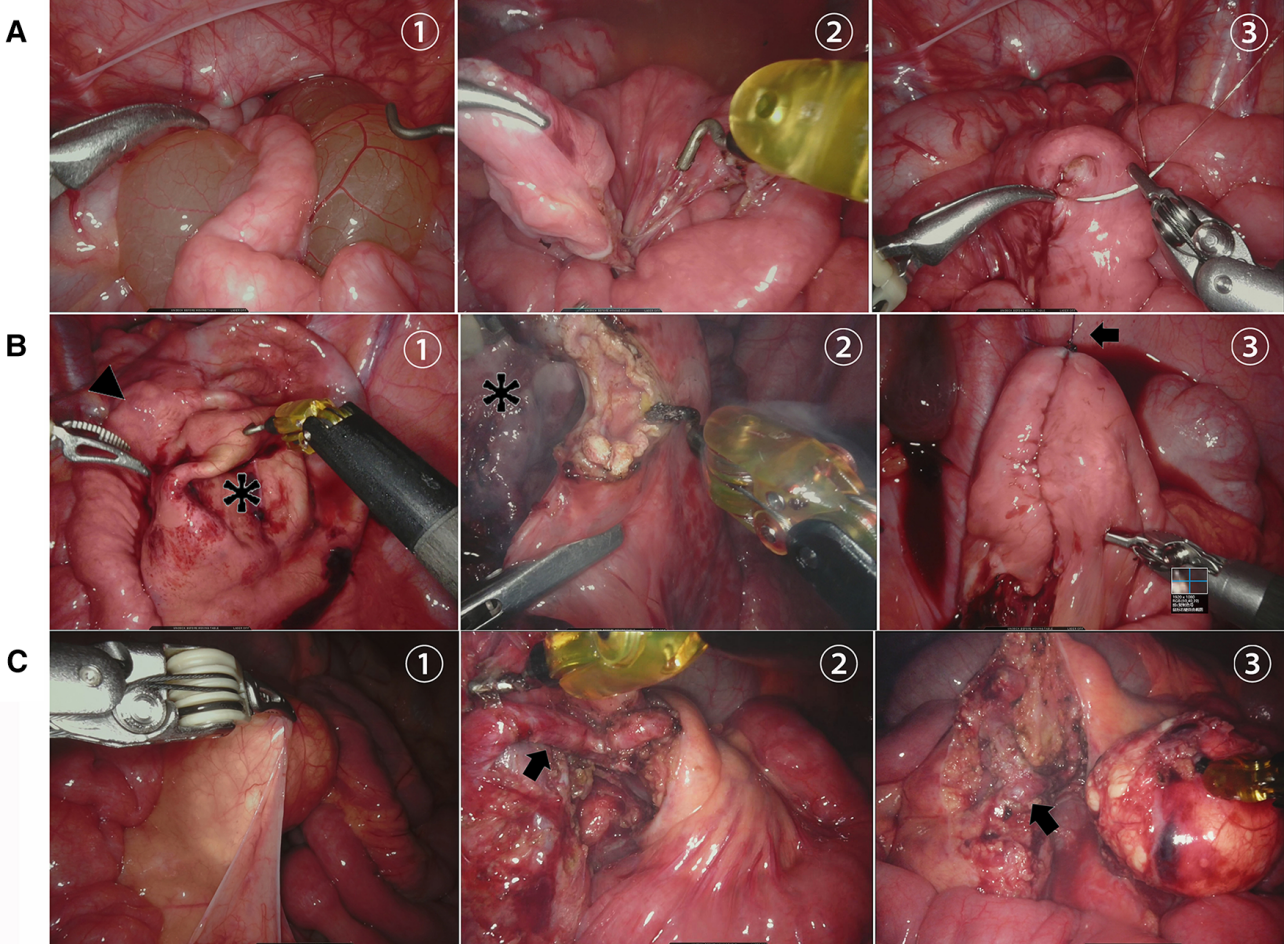
Robot-assisted laparoscopic removal of MCs. (**A**) (1) Serous cyst (asterisk) penetrating through the mesentery of the ileum, (2) complete removal of the cyst without bowel resection, and (3) repair of the mesenteric defect. (**B**) (1) Milky white cyst (asterisk) located at the root of the intestinal mesentery, (2) cyst stripped off the mesenteric vessel, and (3) mesenteric vessels (arrow) preserved intact after cyst resection. (**C**) (1) Huge cyst (asterisk) located in the mesentery of the ileum (arrowhead) after aspiration, (2) cyst removed with segmental bowel resection, and (3) intra-abdominal intestinal anastomosis with the assistance of bowel suspension and traction (arrow).

All patients were operated on by robotic-assisted laparoscopy successfully. All the cysts were completely removed without conversion. Simple cyst excision was performed in eight patients (Figure 4A), including one case of a cyst located at the root of the mesentery and encased mesenteric vessels (Figure 4B). Cyst excision with resection of the involved intestine was required in four cases because the cyst tightly surrounded the mesenteric side of the bowel (Figure 4C). The mean operation time was 106.17 ± 33.74 min (range, 59–170 min).

**Figure 5:**
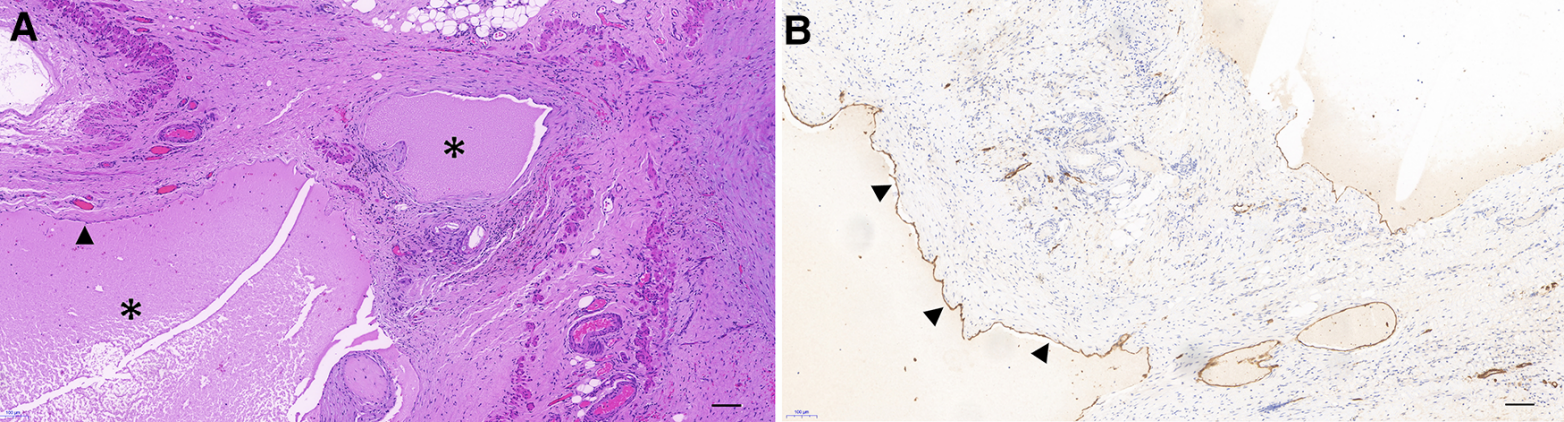
Pathological and immunohistochemical staining of MCs (×100). (**A**) Multiple cysts are lined with a single layer of flattened endothelium (arrowhead), filled with eosinophilic proteinaceous material (asterisk). (**B**) Immunohistochemical staining showing that the endothelium exhibited a positive expression of D2-40. (Bar: 100 μm.)

Postoperative histopathology of HE staining or immunohistochemistry (IHC) was performed routinely, all of which lymphangioma or lymphatic malformations and were positive for D2-40, a marker of the lymphatic epithelium (Figures 5A,B).

All patients were discharged uneventfully, with a mean length of postoperative hospital stay of 7.83 ± 3.33 days (range, 4–15 days). Two patients developed chylous leakage postoperatively and recovered after conservative treatment. No complications of peritoneal sepsis, wound infection, anastomotic leakage, intestinal obstruction, and cyst recurrence were observed during the short- and medium-term follow-up.

## Discussion

MCs are relatively rare benign abdominal lesions, mainly caused by congenital malformation of lymphatic tissue and less commonly by acquired factors, such as infection and trauma. According to the location and morphology of the cyst, MCs are commonly classified into three types (9): broad basal type, dumbbell type, and complicated type. In our series, postoperative pathology revealed mesenteric lymphatic malformation in all cases, and no acquired MCs were found. Intraoperatively, six cases of dumbbell type, one case of complicated type, and five cases of broad basal type were found.

Depending on the site and size of the MCs, various symptoms, including stomach ache, abdominal distension, and abdominal mass, may present, mainly due to the compression of the cyst or association of complications (2). Because of the nonspecific symptoms, there were reports that MCs might not be diagnosed preoperatively (7). In this group, 83.33% (10/12) patients were symptomatic and 66.67% (8/12) patients were with cyst complications. In total, 83.33% (10/12) patients were diagnosed by preoperative ultrasonography, CT, or MRI according to the characteristic imaging manifestations of multilocular and intra- or intercystic separation (10). Only two patients with a single cyst or giant cyst were diagnosed with an abdominal cyst or omental cyst, respectively. Ultrasonography and MRI can clearly delineate the separation of the cyst and have the diagnostic value for MCs, while enhanced CT has more advantages in distinguishing the cyst from surrounding tissues, especially with mesenteric vessels.

Various complications related to MCs like infection, hemorrhage, volvulus, intestinal obstruction, and even malignant transformation have been widely recognized (10–12). In this series, 66.67% (8/12) patients were associated with MC complications, including five cases of infection, two cases of volvulus, and one case of hemorrhage. It was recommended that MCs should be removed as early as possible after diagnosis was confirmed (3, 6, 13). Complete excision is the treatment of choice to avoid the risk of recurrence. However, complete resection might not always be feasible because most of the MCs were multilocular, without a definite margin, and with the potential involvement of major blood vessels and/or visceral organs (14). MCs still carried relatively high recurrence rates after surgery, especially for those located at the root of the mesentery and closely involved in major mesenteric vessels (15). Steyaert et al. reported that in a series of abdominal lymphatic cysts in children, the recurrent rate was 9.5% after open surgery (16). Various assistant treatments were proposed in the case of complete excision failure. Sclerosing agents such as bleomycin, polidocanol, and ethanol were widely accepted as an effective treatment of lymphangioma by eliminating endothelial cells of the cyst. Local injection of sclerosing agents after cyst marsupialization was advocated for the therapy of MCs but with a moderate risk of recurrence (17). Recently, sirolimus has been used to treat lymphatic malformations and achieved good outcomes (18, 19).

Although open surgery is currently the preferred choice of most pediatric surgeons, laparoscopic MC excision is becoming increasingly popular (20, 21). The main methods of laparoscopic approach or laparoscopy-assisted surgery are taking the cyst and the involved bowel loop out of the abdomen through a slightly extended umbilical incision after cystic fluid drainage. Cyst excision, intestinal segment resection, and anastomosis are performed extracorporally (8, 13). Incomplete cyst resection, peritoneal contamination during intestinal resection, and anastomosis are the main reasons impeding the implementation of intra-abdominal laparoscopic MC resection (13). The reported conversion rate of laparoscopic MC resection varied between 6.4% and 33.3% (8, 15). We do not routinely perform cyst aspiration prior to resection to avoid collapse of the cyst, which may result in unclear boundary with the surrounding tissues and incomplete cyst removal and inevitably increase the risk of postoperative recurrence, unless a huge mass obviously limited the intra-abdominal working space and interfered with the visual field of operation. Adequate preoperative intestinal preparation and application of bowel suspension and traction technology can significantly reduce the spillage of intestinal contents and avoid the contamination of the abdominal cavity.

We performed a consecutive of 12 cases of MCs by robotic-assisted laparoscopic surgery successfully, including four cases of intestinal resection and one case of MCs located at the root of the mesentery, without conversion during surgery. An ultrasonic scalpel or electrocoagulation was used to dissect the cyst. Small mesenteric vessels that do not affect the intestinal blood supply were directly transected, while large mesenteric vessels were preserved by peeling the thin cyst wall off the vessels through blunt and sharp separation. Vascular skeletonization of the involved mesenteric vessels might significantly reduce postoperative recurrence. In the case of capsule residue suspected, electrocautery or iodine tincture was used to destroy the epithelium. When the cyst infiltrates the intestinal wall and cannot be easily separated or the impairment of intestinal circulation is observed, intestinal resection and anastomosis are required. No anastomotic leakage and intra-abdominal infection occurred postoperatively, and no recurrence of cysts in the short- and medium-term follow-up was found.

Robotic-assisted laparoscopic surgery has incomparable advantages over conventional laparoscopic surgery to perform MC resection (22). First, the three-dimensional stereoscopic view and 10 times magnification of the operating field provide a clearer surgical vision than open surgery, distinctly identifying the boundary between a cyst and surrounding tissue possible. Second, the EndoWrist surgical instruments with 7 degrees of motion mimic human dexterity and improve control of fine movements, which is helpful to remove the cyst from the bowel or mesenteric vessels delicately and is more suitable for the operation of narrow spaces, especially the cysts located in the lesser sac or the small curvature of the stomach. All the cysts were completely removed by our group, and no marsupialization and assistant treatment methods were applied. The operation time, including the docking procedure in our group, was 59–170 min and was not significantly prolonged as compared with reported laparoscopic or laparoscopic-assisted procedures.

## Conclusions

In conclusion, MCs are relatively rare clinical scenarios. They should be removed promptly once the diagnosis is confirmed to avoid cyst complications. Due to the characteristics of multilocular and fibrous separation, it is not difficult to diagnose MCs preoperatively. Laparoscopic management should be the treatment of choice for most cases of MCs. For uncomplicated mesenteric cysts, laparoscopic cyst excision or cyst excision with bowel resection can be safely performed in children, especially under the robot system. While for complicated MCs, minimally invasive surgery should be selected prudently. When complete removal is unlikely, sclerosing agents therapy and other methods can also be considered.

## Data Availability

The original contributions presented in the study are included in the article/Supplementary Material, further inquiries can be directed to the corresponding author.
